# Factors associated with early newborn care practices in Bangladesh: evidence from Bangladesh Demographic and Health Survey

**DOI:** 10.1007/s43999-023-00027-5

**Published:** 2023-08-09

**Authors:** Md. Saifullah Sakib, Tahmina Ferdous Tanny, Abu Sayeed Ripon Rouf, Mehedi Hasan Manik

**Affiliations:** 1 Department of Statistics, Jagannath University, Dhaka, 1100 Bangladesh; 2 Department of Public Administration, Jagannath University, Dhaka, 1100 Bangladesh; 3 ACI Limited, Dhaka, Bangladesh

**Keywords:** Delivery kit, Newborn, Drying, Skin-to-skin, Delayed

## Abstract

**Aim:**

Immediate care of newborns is essential to scale back the mortality rate. This study tries to search out several aspects of newborn care practices of newborn birth from BDHS 2017-18 data.

**Methods:**

Initially, bivariate analysis is employed to look at the differentials' initial newborn care practices by several selected background variables. The study used a simple and multinomial logistic regression model to identify the important determinants of initial care practices. Besides determinates of the factor, the study also compares the results with the cross-sectional survey data of 2014 and 2011.

**Results:**

The percentage of employing a clean delivery kit during delivery and skin-to-skin contact are 22.8 and 13.7 respectively in Bangladesh in 2017 which is lower than the previous report in 2014. The odds of clean delivery kits and skin-to-skin are higher for educated mothers (OR = 3.30 and OR = 1.74) and in the case of delayed bathing the odds of the Rangpur division (OR = 1.90) are more likely higher compared to the reference category. Besides, the odds of a mother's age above 25 and birth order 3+ are less likely to reference the category for newborn care practices.

**Conclusion:**

Factors identified in early newborn care practices will not only help policy makers undertake a series of interventions for improved newborn health but also ensure good -quality health services.

## Introduction

Initial newborn care has the countless importance for the proper development and healthy lifetime of a baby. Newborn initial care focuses on the employment of a clean delivery kit, skin-to-skin care immediately, bathing delay, prevention of hypothermia, and keeping the newborn warm. At the beginning of birth, newborn health and survival rely on the care given to the newborns. Although newborn care is a necessary element to reduce child mortality, it often does not get optimum attention. Newborns are a permeable group and they require more attention and care [26]. Bangladesh remains still struggling to emerge from poverty. Bangladesh ranks 133rd among nations on the Human Development Index (HDI) as presented in the 2019 Human Development Report [30]. Although Bangladesh has flourished as a development icon after reducing deaths of children below the age of five to satisfy the worldwide target four years prior time, the number of newborns dying in Bangladesh remains very high at 62,000 per annum UNICEF [33] where the under-five mortality rate was 27.3 in 2021 [31]. The prime reasons for newborn deaths in Bangladesh are prematurity, sepsis, and situations arising from complications around delivery. Various reasons can be attributed why the health of the newborn neglects despite the massive mortality rates and why most neonatal deaths are unseen and undocumented. Tinker and Ranson [29] stipulate that, as newborn health closely relates with mothers so newborns have a novel need that demands to be addressed in maternal and child health services. In line with World Health Organization (WHO), poor newborn care practices are the main contributors to neonatal morbidity and mortality in developing countries [13]. A group of practices that reduces newborn morbidity and mortality has been identified as essential and these include clean cord care, drying and wrapping the newborn immediately after delivery and delaying the newborn’s first bath 72 h or more to reduce hypothermia risk, and starting breastfeeding within the primary hour of birth [13, 27, 39].

The speed of neonatal mortality rate is high in Bangladesh 17.5 deaths per thousand live births in 2020 [41]. Maximum neonatal deaths are preventable through simple and cost-effective essential newborn care interventions [18]. In regard to the identification of newborns deaths, UNICEF [32] found almost 40% of mortality makes up for under-five deaths in developing countries. Out of the 3.7 million neonatal deaths and 3.3 million stillbirths every year 98% of all happened in developing countries [9]. There are unhealthy newborn care practices common in developing countries [18]. After birth in newborn cord infections and neonatal tetanus, due to unhygienic cord care, still be the important explanation for neonatal morbidity and mortality [37].

Newborn survival is highly contingent upon hygiene cord care [14]. Factors related to cord infections include unhygienic materials, and lack of hand washing of caregivers and birth attendants [20]. In low and middle-income countries, neonatal hypothermia is a crucial contributing factor for death [11, 21, 38]. Factors related to neonatal hypothermia include low birth weight and prematurity, lack of skin-to-skin contact with the mother, and early breastfeeding [21, 23]. Regarding baby’s first bath, WHO recommends that if cultural tradition demands bathing, this could not be done before 6 h and will be given on the second or third day of life, if the baby is healthy and his/her temperature is normal [38]. Addressing morbidity requires continuous care, which is lacking in many communities that ascertains that neonate care often receive little attention in either maternal or child health programs [22]. The highest deficiency in care often occurs during the critical first week of life when most neonatal and maternal deaths occur, usually at home and with no contact with the formal health sector [12]. Almost 70 percent of all sickness care takes place within the home [25]. It is estimated that 60 percent of newborn deaths occur on the first day of delivery because of asphyxia, 47 percent on the second day for infections, and 81 percent is due to severe infections accordingly. However, triplets have 4 times the chance of dying while it is 5 times for the twins. In addition, low birth weight babies have 8 times the risk of dying and partially breast-fed babies have 4 times the risk of dying [40]. The neonatal mortality rate in developing countries is eight times higher than developed countries [24] while in Sub-Sahara Africa had the very best fatality rate in 2019 at 27 /1000 live births followed by Central and Southern Asia with 24 deaths per 1,000 live births [36]. Under five mortality rates has been remarkably reduced in Bangladesh since its independence from 371.3 deaths in 1971 to 22.614 per 1,000 live births in 2022 [6]. However, maternal mortality within the country decreased by 60% from 2000 to 2017 [7].

Though Bangladesh has made significant progress towards the child and maternal health within the past decades the country yet to develop the overall healthcare system to achieve goal 3 of Sustainable Development Goals (SDGs) [17]. In Bangladesh, newborn care practices do not seem to be commonly employed in the correct way and majority do not have adequate knowledge about the recommended practices of newborns. The National Neonatal Health Strategy and Guidelines for Bangladesh recommend a group of essential newborn care practices: the utilization of a clean delivery kit, skin-to-skin with baby, immediate (within 5 min) drying of the infant, and delaying bathing to 72 h after birth [15]. Most of the previous studies for initial newborn care in Bangladesh conducted on a selected area or community or nation-wide data. For newborn care practices, BDHS has been collecting data since 2007 and 2017–18 DHS survey is the fourth survey to gather information on newborn care. National survey on newborn care has been conducted at four years interval in 2007, 2011, 2014 and 2017–18. There is no such study that has used the latest BDHS 2017–18 data, so this study attempts to identify the current condition of newborn care in Bangladesh. Besides, drawing a comparison with the previous studies the study will have a profound insight about the progress so far achieved in newborn care in Bangladesh.

## Data, variables and method

### Data

Bangladesh Demographic and Health Survey (BDHS) is the 8^th^ health care survey in Bangladesh since it started, conducted by National Institute of Population Research Training (NIPORT) under the Ministry of Health and Family Welfare (MOHFW). The sample for the 2017–18 BDHS is nationally representative and covers the entire population living in non-institutional dwelling units within the country. The survey used an inventory of enumeration areas (EAs) from the 2011 Population and Housing Census of the People’s Republic of Bangladesh, provided by the Bangladesh Bureau of Statistics (BBS), as a sampling frame [2]. The primary sampling unit (PSU) of the survey is an EA with a medium of about 120 households [3]. The 2017–18 BDHS is the fourth DHS survey that collected information on newborn care in Bangladesh. Women (*N* = 2463) who gave birth within the past three years, but did not deliver their last-born child in a health facility, were asked about newborn care practices, including using clean delivery kit, drying, skin-to-skin contact, and bathing of the newborn following birth.

### Variables

Initial care practices of newborns relate to diverse essential care after birth, including clean delivery kit, drying within 0–4 min, skin-to-skin contact, and delayed bathing. To understand newborn care practices, we use the subsequent dependent variables:

- Clean delivery kit used
- Drying
- Skin-to-skin contact
- Delayed bathing

Clean delivery kit used means delivery kits contains a soap to permit the birth attendant to wash her hands before the delivery and before cutting the cord; a plastic sheet for the mother to lie on during the birth; sterile gloves, and a sterilized razor blade to cut the cord; clamps for the cord; cotton towels; and a bag for the placenta [33]. Drying is a necessary care of newborns. Skin-to-skin contact is also important for baby’s self-regulation, which stabilizes the heartbeat and breathing patterns. Seventy-five percent of heart and breathing episodes are reduced using skin-to-skin contact [16]. BDHS has collected the response of skin-to-skin contact twice in the year 2014 [4] and 2017-18. Delayed bathing is generally positive to take care of a baby. Because bathing babies early can increase the chance of hypothermia and therefore the recommended practice of delaying first bath in Bangladesh is 72 hours or more [5].

To access the impact of demographic and socioeconomic variables on newborn care, there are several independent variables used. In this research, the independent variables considered are mother’s age at first birth (categorized into four categories), place of residence (rural, urban), division (Barisal, Chittagong, Dhaka, Khulna, Mymensingh, Rajshahi, Rangpur and Sylhet), religion (Islam, Hinduism, Buddhism and Christianity), wealth index (poor, middle, rich), mother’s education (no education, primary, secondary and higher), birth order (1, 2, 3 +) and gender (male, female) and these are potential factors for newborn care practices in Bangladesh.

### Methods

The 2017–18 BDHS asked mothers who had non-institutional deliveries within the past 3 years about all three components of newborn thermal care: clean delivery kit used during delivery, when the newborn was first dried, whether the newborn was given skin-to-skin care, and when the newborn was first bathed. The chosen dependent and independent variables are described here in extensive form before performing any statistical analysis. Initially, bivariate analysis was performed. After that, logistic regression model has been used to determine the predictors of newborn care practices. Finally, we applied a multinomial logistic regression model for clean delivery kit used, drying, skin-to-skin contact, and delayed bathing called as essential newborn care practices after delivery. Therefore, we have three categories of essential newborn care like i) a minimum of three newborn care practices, ii) two newborn care practices and iii) one practice after delivery of newborns Table 1.

**Table 1:** Description of dependent variables within the study

Variables	Codes
Clean delivery kit used	0 = No
	1 = Yes
Drying	0 = more than 4 min
	1 = within 0–4 min
Skin-to-skin contact	0 = No
	1 = Yes
Delayed bathing	0 = Before 72 h
	1 = 72 h or more

## Results

**Fig. 1:**
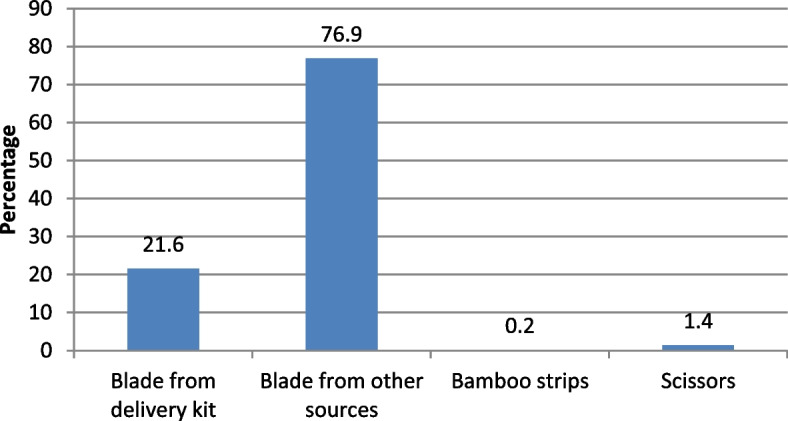
Instrument used to cut the umbilical cord

**Fig. 2:**
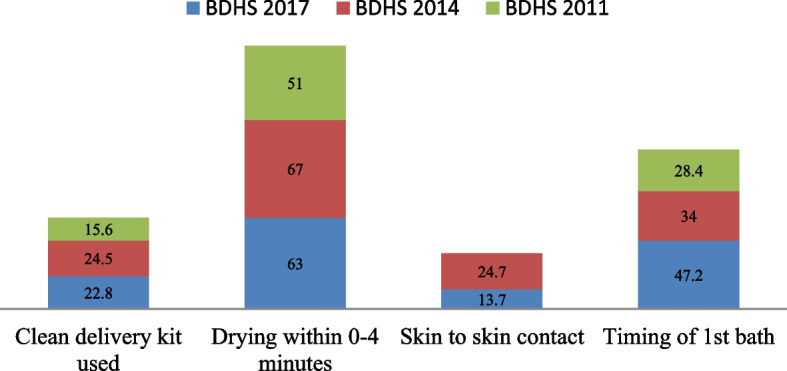
Percetage of care practices in last three BDHSs data

**Fig. 3:**
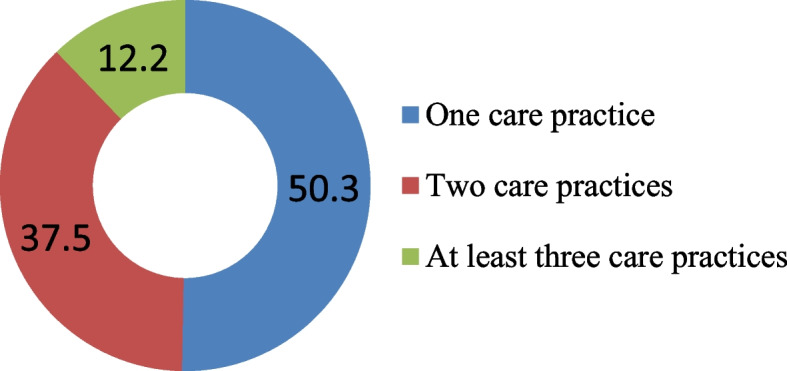
Percentage of essential newborn care practices

Table 2 shows the prevalence of selected newborn care practices by background characteristics. To examine the strength of association of selected dependent variable with the background variables, χ^2^-test also described and presented in Table 2. Among all the age groups increased newborn care practices are found in mothers aged 21–25 at first birth. On the other hand, the rate of skin-to-skin contact after birth is hardly 5.6% for the mother age above 25. For mother’s education, the tendency of clean delivery kit used and skin-to-skin contact increase with the level of education. The percentage of clean delivery kit used during delivery and after births skin-to-skin contact were 36.1 and 18.6 respectively, which is relatively lower for healthy baby. The proportion of wealth index for the rich class was 28.6% for clean delivery kit during delivery, 64.2% for drying baby within 0–4 min and 17.2% for skin-to-skin contact with births while for middle class delayed newborn bath was 45.4%. Among divisions, Rangpur has the highest tendency (32.5%) to use clean delivery kit whereas it is the lowest (18.5%) for Sylhet. Maximum proportion for drying baby within 0–4 min was found in Barisal (65.2%) while the minimum was in Rajshahi division (59.7%). Findings suggest that skin-to-skin contact was the lowest in Rajshahi (7.7%) compared to Rangpur (16.6%) which is the highest in doing so, while the tendency of bathing newborns after 72 h was the lowest in Mymensingh (36.2%). In case of order preference of birth, the first baby had higher percentage for using clean delivery kit, birth order for over three had greater rate of drying within 0–4 min. Although there was slight percentage change in skin-to-skin contact and delayed bathing in birth order. If we consider religion, the rate of using clean delivery kit and delayed bathing was higher in Christian at 28.6% and 85.7% respectively, though the maximum proportion of skin-to-skin contact was in Islam at 14%. Figure 1 shows out of the different instrument used to cut the umbilical cord of a newborn baby 21.6% used blade from delivery kit while the majority (76.9%) have used blade from other sources. In case of trend for baby care after birth, Fig. 2 demonstrates only increasing percentage in timing of first bath from the year 2011 to 2017. Otherwise, in BDHS data of 2011–2017 there were minor variation for using clean delivery kit and drying within 0–4 min during delivery. In terms of skin-to-skin contact the percentage has been reduced from 24.7 in the year 2014 to 13.7 in 2017–18. It is evident from Fig. 3 that half (50.3%) of the total respondents practiced one care while it is 37.5% and 12.2% for two care and three care practices respectively.

**Table 2:** Percentage of most recent non-institutional births in the 3 years preceding the survey by clean delivery kit used, timing of drying, delayed bathing the newborns and percentage of births with skin-to-skin contact

Background characteristics	Clean delivery kit used		Drying		Skin-to-skin contact		Delayed bathing		Number of births (N)
	Yes	*p*-value	Within 0–4 min	*p*-value	Yes	*p*-value	72 h or more	*p*-value	
Mother’s age at 1^st^ birth									
Age below 16	20.5		63.0		14.7		43.0		43
age 16–20	22.8	0.172	62.7	0.08	13.1	0.066	47.1	0.002	1696
age 21–25	26.9		66.4		16.3		55.5		301
age above 25	16.7		47.2		5.6		30.6		36
Mother’s education									
No education	14.6		67.0		11.6		40.8		233
Primary	18.1	0.000	62.8	0.35	12.6	0.012	47.3	0.217	937
Secondary	26.2		62.2		14.2		48.3		1110
Higher	36.1		64.5		18.6		48.1		183
Wealth index									
Poor	19.2		63.2		12.9		47.9		1420
Middle	26.5	0.000	60.7	0.49	11.6	0.0124	45.4	0.639	456
Rich	28.6		64.2		17.2		46.8		587
Region									
Barisal	27.7		65.2		15.8		59.0		170
Chittagong	20.6		63.9		13.0		39.9		560
Dhaka	21.6		62.2		13.9		40.5		538
Khulna	21.7	0.000	61.9	0.95	14.3	0.008	46.0	0.000	175
Mymensingh	21.0		64.1		15.2		36.2		256
Rajshahi	21.0		59.7		7.7		47.6		270
Rangpur	32.5		63.5		16.6		56.5		268
Sylhet	18.5		63.3		12.6		54.4		225
Place of residence									
Urban	25.1	0.059	60.7	0.093	13.7	0.473	45.9	0.251	529
Rural	22.0		63.8		13.5		47.6		1934
Sex of child									
Female	24.1	0.000	61.4	0.057	14.1	0.281	46.9	0.393	1210
Male	21.5		64.6		13.2		47.5		1253
Birth order									
1	27.7		57.8		14.6		45.9		725
2	21.3	0.001	64.5	0.002	12.7	0.556	48.2	0.671	809
3 +	20.2		65.8		13.8		47.3		929
Religion									
Islam	23.0		63.2		14.0		47.1		2301
Hinduism	20.0	0.624	62.1	0.566	11.0	0.296	48.3	0.142	145
Buddhism	10.0		50.0		0.0		30.0		10
Christianity	28.6		42.9		0.0		85.7		7
Total	22.8		63.0		13.7		47.2		2463

From the logistic regression model, we can see how selected component related with demographic and socioeconomic characteristics. Table 3 shows the logistic regression estimates of the women’s background variables on newborn care practices in Bangladesh. From results, using clean delivery kit during delivery and drying newborn within 0–4 min for mother’s age 21–25 is 1.43 (CI: 1.01–2.02) and age above 25 is 1.90 (CI: 0.96–3.77) times more likely than a mother’s age below 16. Besides, births with skin-to-skin contact and timing of a first bath of babies for mother’s age above 25 are 0.34 (CI: 0.08–1.46) and 0.58 (CI: 0.28–1.21) times lower compared to mother’s age below 16. The odds of using clean delivery kit, skin-to-skin contact after births, and delayed bathing are greater for higher educated mother compared to no educated mother, respectively. The odds ratio of middle- and rich-income class is 1.52 and 1.69 times for using clean delivery kit and for skin-to-skin contact after births rich class is 1.40 times more likely to poor income class significantly. Among region, the odds of using clean delivery kit during delivery women from Barisal and Rangpur are 1.39 (CI: 0.96–2.02) & 1.74 (1.19–2.56) and the odds of timing first bath of newborn women from Barisal, Rangpur & Sylhet are 2.11 (CI: 1.53–2.92), 1.90 (CI: 1.36–2.66) & 1.75 (CI: 1.29–2.37) times higher compared to women from Dhaka division. However, the odds of drying newborn within 0–4 min and skin-to-skin contact with baby after birth women from Barisal and Rajshahi are 0.88 and 0.52 times lower compared to Dhaka, respectively. In case of birth preference, the odds of first baby get all newborn care practices compared to other birth order. As a religious prospectus, the odds ratio of using clean delivery is 0.36 and for delayed bathing is 0.48 of Muslim compared to Buddhism. For being Christianity, the odds of using clean delivery kit, drying baby within 0–4 min and timing of first bath are more likely compared to Islam, respectively.

**Table 3:** Regression parameter estimates (β) and odds ratio obtained using logistic regression model for clean delivery kit used, drying, skin-to-skin contact and delayed bathing of newborns separately

Background characteristics	Clean delivery kit used		Drying		Skin-to-skin contact		Delayed bathing	
	Odds Ratio	95% C.I	Odds Ratio	95% C.I	Odds Ratio	95% C.I	Odds Ratio	95% C.I
Mother’s age at 1^st^ birth								
Age below 16 (Ref)								
age 16–20	1.14	0.88–1.48	1.01	0.81–1.26	0.88	0.65–1.19	1.18	0.95–1.46
age 21–25	1.43	1.01–2.02	0.86	0.63–1.17	1.13	0.75–1.70	1.65	1.23–2.22
age above 25	0.78	0.31–1.92	1.90	0.96–3.77	0.34	0.08–1.46	0.58	0.28–1.21
Mother’s education								
No education (Ref)								
Primary	1.29	0.86–1.94	1.20	0.88–1.63	1.10	0.70–1.72	1.30	0.97–1.74
Secondary	2.08	1.41–3.06	1.23	0.91–1.66	1.27	0.82–1.96	1.36	1.02–1.81
Higher	3.30	2.06–5.29	1.11	0.74–1.68	1.74	1.00–3.01	1.35	0.91–1.99
Wealth index								
Poor (Ref)								
Middle	1.52	1.19–1.95	1.12	0.90–1.38	0.89	0.64–1.23	0.90	0.73–1.12
Rich	1.69	1.35–2.11	0.96	0.78–1.17	1.40	1.08–1.83	0.95	0.79–1.16
Region								
Dhaka (Ref)								
Barisal	1.39	0.96–2.02	0.88	0.63–1.22	1.17	0.74–1.83	2.11	1.53–2.92
Chittagong	0.94	0.66–1.35	0.97	0.71–1.31	0.93	0.60–1.43	0.97	0.72–1.32
Khulna	1.01	0.64–1.56	1.01	0.69–1.47	1.03	0.61–1.75	1.25	0.87–1.81
Mymensingh	0.96	0.66–1.41	0.92	0.67–1.27	1.11	0.71–1.73	0.83	0.60–1.14
Rajshahi	0.97	0.63–1.47	1.11	0.78–1.58	0.52	0.29–0.93	1.33	0.94–1.89
Rangpur	1.74	1.19–2.56	0.94	0.67–1.33	1.23	0.77–1.96	1.90	1.36–2.66
Sylhet	0.82	0.56–1.19	0.95	0.69–1.30	0.89	0.57–1.40	1.75	1.29–2.37
Place of residence								
Urban (Ref)								
Rural	0.83	0.68–1.04	1.13	0.95–1.37	1.01	0.77–1.32	1.07	0.89–1.29
Sex of child								
Female (Ref)								
Male	0.86	0.95–1.39	1.14	0.96–1.34	0.93	0.88–1.36	1.02	0.83–1.15
Birth order								
1 (Ref)								
2	0.70	0.56–0.89	0.75	0.61–0.93	0.85	0.64–1.14	1.09	0.89–1.34
3 +	0.66	0.53–0.83	0.71	0.58–0.87	0.93	0.71–1.23	1.05	0.86–1.28
Religion								
Islam (Ref)								
Hinduism	0.84	0.55–1.27	1.05	0.75–1.49	0.77	0.45–1.30	1.04	0.74–1.46
Buddhism	0.36	0.05–2.85	1.57	0.45–5.48	-	-	0.48	0.12–1.86
Christianity	1.32	0.26–6.85	2.12	0.47–9.56	-	-	6.74	0.81–9.14

*C. I* Confidence Interval

The results of the multinomial logistic regression model are presented in Table 4. For mother age at first birth, applying ‘one newborn care practice’ compared to ‘at least three care practices’, mother’s age above 25 compared to mother’s age below 16 are more likely to practice one newborn care practice, given the other variables in the model are held constant. For applying ‘one care’ and ‘two care’ compared to ‘at least three cares’ secondary and higher educated mother are less likely compared to no educated mother to practice one and two care respectively [$exp(\beta )$=0.807, CI: 0.501–1.301; $exp\left(\beta \right)=$ 0.655, CI: 0.404–1.062; $exp(\beta )$=0.535, CI: 0.291–0.982 & $exp(\beta )$=0.594, CI: 0.323–1.095], given the other variables in the model are held constant. For wealth index, one care practice compared to at least three cares rich class compared to poor class is less likely to practice one care [$exp\left(\beta \right)=0.883, \mathrm{CI}:0.492-0.892 ].$ In case of gender, two care compared to at least three care males compared to female are less likely to practice two cares [$exp\left(\beta \right)=0.766, \mathrm{CI}: 0.590-0.995]$. In region, the comparison will be Dhaka to others division. One newborn care practice and two care practices compared to at least three care practices, Mymensingh [exp(β) = 0.606], Rangpur [exp(β) = 0.548], Barisal [exp(β) = 0.652], Khulna [exp(β) = 0.658], and Rangpur [exp(β) = 0.391] compared to Dhaka, are less likely to use one newborn care practice and two care practices, given the other variables in the model are held constant. Finally, one care practice compared to three care practices rural compared to urban for residence is less likely [exp(β) = 0.674] and for Christianity compared to Islam is more likely [exp(β) = 1.478] to practice one newborn care after birth.

**Table 4:** Multinomial logistic regression model of essential newborn care practices

Background characteristics	One newborn care practice		Two newborn care practices	
	$exp(\beta )$	95% CI	$exp(\beta )$	95% CI
Mother’s age at 1^st^ birth				
Age below 16 (Ref)				
age 16–20	1.088	0.777–1.522	1.057	0.748–1.494
age 21–25	0.906	0.577 -1.423	0.804	0.503–1.286
age above 25	2.093	0.513–6.199	1.317	0.358–4.842
Mother’s education				
No education (Ref)				
Primary	1.146	0.700–1.876	0.875	0.531–1.444
Secondary	0.807	0.501–1.301	0.655	0.404–1.062
Higher	0.535	0.291–0.982	0.594	0.323–1.095
Wealth index				
Poor (ref)				
Middle	0.837	0.597–1.173	0.918	0.648–1.301
Rich	0.663	0.492–0.892	0.813	0.599–1.103
Sex of child				
Female (ref)	-		-	
Male	0.850	0.660–1.094	0.766	0.590–0.995
Region				
Dhaka (Ref)	-		-	
Barisal	0.833	0.566–1.623	0.652	0.413–1.212
Chittagong	0.874	0.542–1.454	0.769	0.472–1.279
Khulna	0.773	0.446–1.436	0.658	0.364–1.201
Mymensingh	0.606	0.419–1.163	0.710	0.467–1.297
Rajshahi	1.384	0.796–2.658	0.879	0.477–1.654
Rangpur	0.548	0.388–1.075	0.391	0.256–0.736
Sylhet	1.247	0.837–2.391	0.805	0.506–1.488
Place of residence				
Urban(ref)	-		-	
Rural	0.674	0.727–1.304	0.916	0.678–1.238
Birth order				
1(ref)				
2	1.223	0.889–1.682	1.213	0.872–1.687
3 +	1.123	0.828–1.524	1.131	0.825–1.550
Religion				
Islam (Ref)				
Hinduism	1.173	0.684–2.010	0.893	0.505–1.580
Buddhism*	-	-		-
Christianity	1.478	0.177–12.33	-	-

*C. I* Confidence Interval

Reference category: At least three care practices

Buddhism* sample sizes were too small

## Discussion

Our study showed that drying within 0–4 min (63%), skin-to-skin contact (13.7%) and timing of first bath (47.2%) to the newborns were almost just like the national survey of Bangladesh [3]. Just in case of using clean delivery kit during delivery (22.8%) which were also similar with DHS analytical studies (less than 25%) [34]. The findings of this study show that the non-institutional delivery providers must be educated on harmful effects of using traditional materials on the cord stump with emphasis on essential care practices. In some countries, timing of the primary bath, the newborn is placed on the ground without drying and wrapping and given a shower immediately considering vernix and blood on skin to be dirty [10, 19]. Thus, activity may cause hypothermia, and delay in putting the newborn on to the mother’s breast. That’s why, to prevent forestall hypothermia, it’s important to stay the baby pleasantly warm. Recommendations to lessen forestall hypothermia in infant including delivery in an exceedingly warm room, immediate drying and wrapping with dry warm cloths, skin-to-skin contact with mother for the primary few hours after birth, and early breastfeeding [39]. Although WHO recommends delaying the primary bath until after 24 h, Bangladesh newborn care policy and guideline suggests delaying the first bath for 72 h [15]. Our study showed timing of first bath up to 72 h was 47.2% and there has been also regional variation which is analogous to other studies conducted in Asia and Africa [10, 19, 28, 35]. Baqui AH et al. [8] found that in south Asia the mother and her baby become polluted or dirty after birth which results from the birthing process and it’s perceived that the baby’s risk of dying is reduced by bathing. The determinants of newborn look after in regard to drying within 0–4 min and delayed bathing have increased among the educated mothers and in some regions of the country from a previous study conducted by [26].Our study is different from other studies in regard to religion, as it shows the Christianity has higher odds for baby initial care [18].

In Bangladesh, mother’s age at first birth, education, religion, wealth index and birth order are important issue for newborn care practices. During this study, some demographic variables are found having significant effect on newborn care practices of using clean delivery kit, drying, skin-to-skin contact and timing of first bath. This study about newborn care issues is comparatively first research in Bangladesh using BDHS data 2017–18. This topic has gained its significance for the high portion of the infant and under five years mortality and to comply with the international guidelines towards eliminating such child death rate in Bangladesh. The international communities focus and the pledge of Bangladesh government on the fulfillment of SDG goal number 3 within 2030 have made newborn care practices a matter of utmost consideration. Without a considerable decrease within the mortality and morbidity of newborns, the kid health indicators can-not be improved. Community awareness of neonatal complications and care-seeking practices can significantly improve newborn health. There is an urgent need to strengthen routine screening by healthcare providers at the community level to dispel myths and eliminate reliance on traditional healers [1]  There are some research studies on some areas specially using clean delivery kit during delivery and skin-to-skin contact of newborn health like the determinants of newborn care practices, mortality, and morbidity and future research on newborn health should concentrate on the interrelationships between various factors related to newborn care.

## Conclusion

All newborn care techniques are critical for lowering morbidity and death in Bangladesh. This study looked at the relationship between fundamental newborn care practices and several demographic and socioeconomic characteristics in Bangladesh. A newborn infant may play a vital part in keeping every member of a happy family smiling. 

 According to (BDHS, 2017–18) in Bangladesh half of delivery occurred at home compared to health center, so professional person and traditional birth attendant should be qualified and given adequate training especially in remote areas where hospital access is limited. Superstition and unscientific methods of newborn practices should be eliminated from villages by means of awareness building programs by voluntary and Non-Government Organizations (NGOs).

As a result, the importance of newborn care practices should be realized to scale back morbidity and mortality on effective implementation of widespread teaching programs for the community and home delivery attendants using clean delivery kit, skin-to-skin contact, delayed bathing and WHO-recommended initial care to benefit newborn survival in Bangladesh.
